# Genome-wide association study of pre-harvest sprouting resistance in Chinese wheat founder parents

**DOI:** 10.1590/1678-4685-GMB-2016-0207

**Published:** 2017-07-10

**Authors:** Yu Lin, Shihang Liu, Yaxi Liu, Yujiao Liu, Guoyue Chen, Jie Xu, Mei Deng, Qiantao Jiang, Yuming Wei, Yanli Lu, Youliang Zheng

**Affiliations:** 1 Triticeae Research Institute, Sichuan Agricultural University, Wenjiang, Chengdu, P.R. China; 2 Maize Research Institute, Sichuan Agricultural University, Wenjiang, Chengdu, P.R. China

**Keywords:** General linear model, linkage disequilibrium, marker-trait associations, mixed linear model, seed dormancy

## Abstract

Pre-harvest sprouting (PHS) is a major abiotic factor affecting grain weight and quality, and is caused by an early break in seed dormancy. Association mapping (AM) is used to detect correlations between phenotypes and genotypes based on linkage disequilibrium (LD) in wheat breeding programs. We evaluated seed dormancy in 80 Chinese wheat founder parents in five environments and performed a genome-wide association study using 6,057 markers, including 93 simple sequence repeat (SSR), 1,472 diversity array technology (DArT), and 4,492 single nucleotide polymorphism (SNP) markers. The general linear model (GLM) and the mixed linear model (MLM) were used in this study, and two significant markers (*tPt-7980* and *wPt-6457*) were identified. Both markers were located on Chromosome 1B, with *wPt-6457* having been identified in a previously reported chromosomal position. The significantly associated loci contain essential information for cloning genes related to resistance to PHS and can be used in wheat breeding programs.

## Introduction

In Chinese wheat breeding programs, the crossing parents, known as “founder parents,” are wheat varieties or germplasm lines with excellent properties, such as resistance to numerous biotic and abiotic stresses, high combining ability, and strong heritability of superior agronomic traits, that are used for the development of improved cultivars with wide application value (Zhuang, 2003; Chen *et al.*, 2013). Over the past seven decades, more than 2,000 wheat (*Triticum aestivum* L.) cultivars have been released through pedigree selection programs in China. Most of these, however, can be traced back to only 16 ancestral founder parents (Zhuang, 2003; Li *et al.*, 2012). Founder parents have been used in multiple studies on genetic diversity (Wang *et al.*, 2007; Li *et al.*, 2008; 2012), quantitative trait loci (QTL) mapping (Lin *et al.*, 2006; Su *et al.*, 2006; Ma *et al.*, 2007) and association studies (Breseghello and Sorrells, 2006a; Crossa *et al.*, 2007; Xiao *et al.*, 2011).

PHS is the germination of grains within a physiologically mature wheat spike before harvest (Groos *et al.*, 2002; Jaiswal *et al.*, 2012). PHS in wheat (*Triticum aestivum* L.) is the result of the early break in seed dormancy under humid and wet conditions prior to harvest (Rikiishi and Maekawa, 2010; Kulwal *et al.*, 2012), causing significant losses in grain weight and end-product quality. Percent germination (PG) is a variable commonly used to characterize the resistance to PHS based on seed dormancy (Knox *et al.*, 2005, 2012; Rasul *et al.*, 2009; Singh *et al.*, 2010; Kumar *et al.*, 2015). Previous studies reported that almost all 21 chromosomes of hexaploid wheat contain QTL for PHS resistance (Roy *et al.*, 1999; Zanetti *et al.*, 2000; Mares and Mrva, 2001; Flintham *et al.*, 2002; Kulwal *et al.*, 2005; Chen *et al.*, 2008; Fofana *et al.*, 2009; Kulwal *et al.*, 2010; Kim *et al.*, 2014; Somyong *et al.*, 2014). One major QTL *TaPHS1* mapped on Chromosome (Chr.) 3AS has been cloned (Nakamura *et al.*, 2011; Liu *et al.*, 2011), whereas another major QTL on Chr. 4AL has been fine mapped with SNP markers (Cabral *et al.*, 2014; Barrero *et al.*, 2015;Lin *et al.*, 2015).

AM, also known as LD mapping, relies on existing natural populations or specially designed populations to overcome the constraints of linkage mapping (Pasam *et al.*, 2012). AM is a powerful tool for resolving complex trait variations and identifying different loci and/or novel and superior alleles in natural populations (Zhu *et al.*, 2008). In recent years, association studies have been extensively used to discover and validate QTL or genes for important traits and map candidate genes in many crop plants. In wheat, different association panels have been used in many AM studies to identify loci controlling agronomic (Breseghello and Sorrells, 2006b; Crossa *et al.*, 2007; Neumann *et al.*, 2007; Bordes *et al.*, 2013) and quality (Ravel *et al.*, 2009; Bordes *et al.*, 2011) traits, including PHS resistance (Kulwal *et al.*, 2012).

In this study, we aimed to: 1) investigate marker-trait associations (MTAs) for PHS resistance based on a whole-genome AM approach using seed dormancy variables in combination with SSR, DArT, and SNP markers in a core collection of 80 Chinese wheat founder parents; 2) estimate the extent of LD using SNP markers for the A, B and D genomes and the whole genome; and 3) identify candidate genes controlling PHS resistance. The identified significantly associated loci might contain essential information for cloning genes related to PHS resistance and be useful in wheat breeding programs.

## Material and Methods

### Phenotyping

Eighty founder parents and their derivatives, collected by the Triticeae Research Institute of Sichuan Agricultural University, were used to identify PHS resistance. The experimental materials were grown in a randomized complete block design over two growing seasons (2012 and 2013) in Wenjiang and Ya'an (12WJ, 13WJ, 12YA, and 13 YA) and one growing season (2014) in Chongzhou (14CZ). In each of the five environments, thirty spikes were harvested from each genotype at the late dough stage of ripening (Yang *et al.*, 2007; Xia *et al.*, 2009), hand-threshed to avoid damaging the embryos, and sterilized with 1% (v/v) NaClO. Three sets of fifty seeds from each genotype were then placed into Petri dishes with one layer of filter paper and 10 mL of distilled water in each environment. Seeds without damage were stored in a shaded, cool room before being germinated at 20 °C for seven days. Germinated seeds were removed daily and counted. PHS resistance was assessed by PG based on seed dormancy (Knox *et al.*, 2005; 2012; Rasul *et al.*, 2009; Singh *et al.*, 2010; Kumar *et al.*, 2015). PG (mean values of three repetitions in each environment) and the overall mean values of five environments were analyzed. To eliminate environmental impact from the analysis, the best linear unbiased prediction (BLUP) was also used (Piepho *et al.*, 2008). BLUP was calculated across the five environments using the MIXED procedure in SAS 8.1 (SAS Institute, Cary, NC, USA). Broad-sense heritability was defined as H = VG/(VG + VE), where VG and VE are estimates of genetic and environmental variance, respectively (Smith *et al.*, 1998).

Analysis of variance (ANOVA) was performed using SAS 8.1 (SAS Institute), whereas descriptive statistics and Pearson's correlation using SPSS 20.0 (IBM Corp., Armonk, NY, USA).

### Genotyping

Three types of molecular markers, SSRs, DArTs, and SNPs, were used in this study. DNA was extracted from young seedlings using the CTAB method (Saghai-Maroof *et al.*, 1994) and then, sent to Triticarte Pty. Ltd. (Canberra, Australia) for whole-genome profiling using DArT markers. The panel was also genotyped using the Illumina 9K iSelect SNP chip assay (Cavanagh *et al.*, 2013). Both DArT and SNP markers were filtered to contain < 10% missing values and a minor allele frequency (MAF) threshold > 5%. An additional set of 93 SSR markers distributed across the wheat genome was also screened against the genome-wide association study (GWAS) population. For SSR analysis, PCR was performed as described by Sood *et al.* (2009). PCR products were separated in 8% polyacrylamide gels and visualized by silver staining.

### Population structure

Population structure was estimated with a set of 4,492 SNP markers with an MAF threshold > 0.05 using STRUCTURE 2.3.3, which implements a model-based Bayesian cluster analysis (Pritchard *et al.*, 2000; Wang *et al.*, 2013). Five runs of STRUCTURE were performed with a *K* set between 1 and 10 using the admixture model with 20,000 replicates for burn-in and 20,000 replicates during analysis. The optimal *K*-value was determined using the delta *K* method (Evanno *et al.*, 2005).

### LD analysis

LD estimates and significant differences between 3,285 SNPs with genetic distances based on the Illumina 9K SNP consensus map (Cavanagh *et al.*, 2013) were calculated in TASSEL 3.0. The LD squared allele-frequency correlation (*r* ^2^), which contains both mutational and recombination history, was evaluated for linked/syntenic and unlinked loci (p < 0.05). LD decay scatter plots were generated using *r* ^2^ and the genetic map distance between markers. Non-linear regression equations were performed for LD decay distances.

### Association analysis

Population structure (*Q* matrix) from STRUCTURE was used as covariate for the GLM and MLM, and a marker-based kinship matrix (*K*) obtained using TASSEL was used in the MLM. MTAs were calculated in TASSEL using: i) the GLM with a *Q* matrix and ii) the MLM with a *Q* matrix and kinship. PG from the five environments, mean values, and BLUP values were used in the analysis. All models used 6,057 informative markers (93 SSRs, 1,472 DArTs, and 4,492 SNPs) with a MAF threshold > 0.05. A Bonferroni-corrected threshold at α = 1 was used as the cutoff (Yang *et al.*, 2014). For 6,057 markers, the Bonferroni-corrected *p*-value threshold at α = 1 was 1.65 × 10^−4^ with a corresponding -log *p*-value of 3.782. Significant markers were visualized with a Manhattan plot drawn in R 3.0.3 (R core team, Vienna, Austria). Important *p*-value distributions (observed *vs.* cumulative *p*-values on a -log_10_ scale) were displayed on a quantile-quantile plot drawn in R. To find candidate genes, flanking genes, and trait-related proteins, we performed a Basic Local Alignment Search Tool (BLAST) search against the International Wheat Genome Sequencing Consortium database (IWGSC) using the marker sequences. A BLASTN search against the National Center for Biotechnology Information (NCBI) database was performed using 5 kb of the best survey sequence around the significant marker from IWGSC BLAST results, and genes from the best hits were listed.

## Results

### Phenotypic evaluation

**Figure 1:**
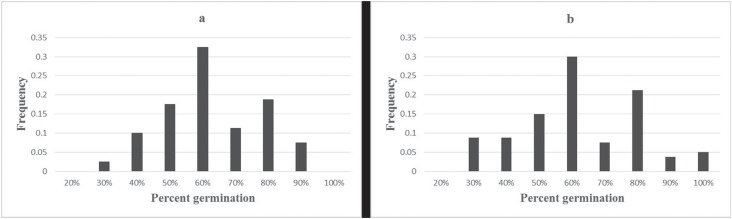
Frequency histograms (a) of BLUPs in 80 Chinese wheat founder parents, (b) of mean values in 80 Chinese wheat founder parents.

PG was significantly different (p < 0.001) among genotypes, environments, and genotype-environment interactions with a heritability of 0.76 (Table 1). Descriptive statistics for the 80 founder parents are shown in Table 2. Pearson's correlation was used to investigate PG across years, as well as BLUP and mean values (Table 3). Significant correlations were observed between all PG values, except for those of 12WJ and 13YA. BLUP and mean values were also highly correlated with the data from the five environments (Table 3). Frequency histograms of BLUP and mean values of the 80 founder parents are shown in Figure 1.

**Table 1:** Variance analysis for percent germination (PG) in five environments.

Variable	DF	Sum of squares	Mean square	F-value
Environments	4	31.19	7.80	892.13***
Replications	5	1.65	0.33	37.72***
Genotypes	79	22.69	0.29	32.86***
G × E	316	18.09	0.06	6.72***

Abbreviation: DF, degrees of freedom.

*** Significant at p < 0.001.

**Table 2:** Descriptive statistics for percentage germination (PG) of 80 founder genotypes.

Environment	Mean	SD	Min	Max	CV
12WJ	22.46%	0.23	0.00%	97.00%	103.74%
12YA	41.99%	0.28	0.00%	99.00%	67.76%
13WJ	90.86%	0.13	27.00%	100.00%	14.33%
13YA	82.68%	0.22	0.00%	100.00%	26.26%
14CZ	49.33%	0.31	1.00%	100.00%	63.82%

Abbreviation: 12WJ, Wenjiang 2012; 12YA, Ya'an 2012; 13WJ, Wenjiang 2013; 13YA, Ya'an 2013; 14CZ, Chongzhou 2014; CV: coefficient of variation; Min, minimum; Max: maximum; SD, standard deviation.

**Table 3:** Correlation coefficients for percent germination (PG).

	PG-12WJ	PG-12YA	PG-13WJ	PG-13YA	PG-14CZ	Mean
PG-12YA	0.604**					
PG-13WJ	0.430**	0.351**				
PG-13YA	0.167	0.344**	0.305**			
PG-14CZ	0.654**	0.680**	0.556**	0.436**		
Mean	0.766**	0.839**	0.630**	0.581**	0.903**	
BLUP	0.722**	0.833**	0.626**	0.580**	0.912**	0.995**

** significant at p < 0.01.

Abbreviation: 12WJ, Wenjiang 2012; 12YA, Ya'an, 2012; 13WJ, Wenjiang 2013; 13YA, Ya'an 2013; 14CZ, Chongzhou, 2014; BLUP, the best linear unbiased prediction.

### Population structure

A set of 4,492 SNP markers was used to estimate the underlying population structure. Delta *K* declined after *K* = 3 and again increased. Using *K* = 3 as inferred by delta *K*, the population was divided into sub-population (Subp)1, Subp 2, and Subp 3. Based on their pedigrees, Subp 1 included the founder parent Fan 6 and its derivatives; Subp 2 included 23 founder parents and their derivatives; whereas Subp 3 only included 11 founder parents.

### LD analysis

**Figure 2:**
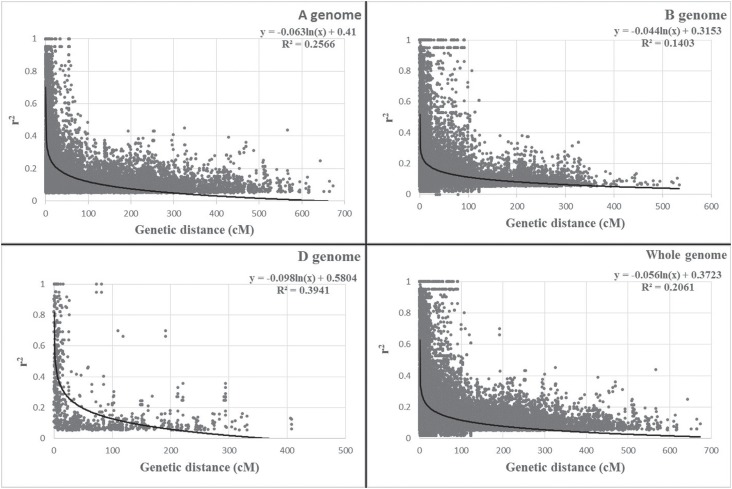
Scatter plots of significant *r* ^2^ values and genetic distance (cM) (p < 0.05) of locus pairs on A, B, D, and whole genomes in 80 Chinese wheat founder parents.

The extent of LD was estimated using SNP data (1,547 loci from the A genome, 1,510 loci from the B genome, and 228 from the D genome). Linked and unlinked locus pairs were detected among the 80 founder parents (p < 0.05). The map distance for which LD fell below the *r* ^*2*^ threshold of 0.3 was determined. This is a frequently used LD threshold in GWAS (Ardlie *et al.*, 2002; Shifman *et al.*, 2003; Khatkar *et al.*, 2008; Maccaferri *et al.*, 2015). The LD decay distances *(r* ^2^ = 0.3) were approximately 1.94, 5.73, 1.42, and 17.48 cM for the whole genome and the A, B, and D genomes, respectively (Figure 2). The D genome had the most significant LD, whereas the B genome had the least significant LD.

### Significant MTAs for PHS resistance

The Bonferroni-corrected threshold (-log p > 3.782, α = 1) was used as a cutoff (Yang *et al.*, 2014) to identify MTAs. A total of 68 significant markers was detected in the five environments by the GLM, with a phenotypic variation of 14.43–30.51% (Table S1; Figures S1–S5). Based on the Illumina 9K SNP consensus map (Cavanagh *et al.*, 2013) and the Triticarte consensus map 3.0 (Chrom_Wheat_ConsensusMap_version_3; Triticarte Pty. Ltd.), most of the significant markers were mapped on Chrs. 1A, 1B, 2A, 2B, 3A, 4A, 4B, 5A, 5B, 6A, and 7A, whereas 10 markers remained unmapped. The significant markers were distributed on all the seven chromosomes of the A genome, but none on the D genome. Besides, the DArT marker *wPt-4301* was significant in both 12WJ and 14CZ. In the MLM, three DArT makers were detected with a phenotypic variation of 21.19–36.82% (Table 4), and two of these markers mapped on Chr. 1B (104.00 cM and 365.57 cM) were also significant in the GLM (Table 4; Table S1; Figures S1–S5).

**Table 4:** Markers showing significant association with pre-harvest sprouting resistance in the mixed liner model.

Environment^a^	Marker^b^	Locus	Locus position (cM)	-Log_10_^(*p*)^	Marker R^2^ (%)
13YA	*wPt-668205*	1A	473.36	3.92	21.19
13WJ	*wPt-6457*	1B	104.00	4.47	36.82
13WJ	*tPt-7980*	1B	365.57	5.34	29.94

a 13WJ, Wenjiang 2013; 13YA, Ya'an 2013.

b significant at the threshhold of -log_10_^(*p*)^ = 3.782.

c the Chromosome and position of DArT markers were from the Triticarte consensus map 3.0 (http://www.triticarte.com.au/).

BLUP and mean values were also calculated for the five environments and analyzed to interpret the results (Table S1; Figures S6, S7). In the GLM, 11 and 10 significant markers were detected using BLUP and mean values, respectively. Eight of these markers were detected using both BLUP and mean values and mapped on Chr. 2B, 3A, 3D, and 5A. However, no significant markers were found by the MLM using BLUP or mean values.

### Putative candidate genes from best hits of significant loci

Based on BLAST search against IWGSC and NCBI using the marker sequences, numerous putative/flanking genes were identified from the best hits of significant loci (Table S1). The identified candidate genes were roughly divided into groups based on the type of encoded proteins. The first group included genes encoding enzymes such as *Acc-1* (Chalupska *et al.*, 2008), *Acc-2* (Chalupska *et al.*, 2008), *CAC3* (Ke *et al.*, 2000), starch synthase I gene (Li *et al.*, 1999), *UBA1* (Hatfield *et al.*, 1990), and waxy gene (Takeuchi T, Sato M, Suzuki T, Yoshimura Y, Nakamichi K, Kobayashi S, Nishimura T, Ikenaga M and Sato N. unpublished). The second group included genes encoding regulatory proteins such as *vp1D* (McKibbin *et al.*, 2002), *Rht-A* (Wu *et al.*, 2013), *Rht-B* (Wu *et al.*, 2013), *VRN3* (Yan *et al.*, 2006), *VRN-A1* (Ivanicova *et al.*, 2016), *VRN-B1* (Guedira M, Xiong M, Johnson J, Marshall D and Brown-Guedira G. unpublished), and *PRR73* (Cockram *et al.*, 2012). The third group included genes encoding transport proteins such as *AACT1* (Silva-Navas *et al.*, 2012) and *HAK11* (Banuelos *et al.*, 2002). The fourth group included genes encoding proteins related to stem rust resistance, such as *Adf2* (Brueggeman *et al.*, 2008), and the fifth group included genes encoding other proteins such as *AIP2-1* (Gao DY, Ma YZ, Xia LQ and Xu ZS. unpublished), GRMZM2G043657-like gene (Jiao *et al.*, 2012), *Hox-1* (Wicker *et al.*, 2009), *NP30_C3* (Zang *et al.*, 2011), *NP35_C3* (Zang *et al.*, 2011), and *Ty3* (Hudakova *et al.*, 2001).

## Discussion

LD decay distance is important in genome-wide association studies. In the present study, LD decay distance indicated the high marker density and precision of association mapping. Many different LD decay distances have been previously reported in wheat. Chao *et al.* (2007) reported genome-wide LD estimates of less than 1 cM for genetically linked locus pairs with *r* ^2^ < 0.2 (p < 0.01) and less than 10 cM between loci among 43 elite U.S. wheat cultivars using 242 genomic SSRs; Cormier *et al.* (2014) reported LD decay distances of 1.12 cM for the whole genome and 0.52, 0.70, and 2.14 cM for the A, B, and D genomes, respectively among 214 European elite varieties using 23,603 genome-wide distributed SNPs; and Maccaferri *et al.* (2015) reported a LD decay distance of 1.6 cM *(r* ^2^ = 0.3) for the whole genome among 1,000 spring wheat genotypes using 4,585 SNPs. In the present study, the LD decay distance *(r* ^2^ = 0.3) was approximately 1.94 cM for the whole genome and 5.73, 1.42, and 17.48 cM for the A, B, and D genomes, respectively (Figure 2). The LD values differed significantly among the three wheat genomes, whereas the D genome had the greatest LD, similar to that reported in previous studies (Cormier *et al.*, 2014; Edae *et al.*, 2014).

**Figure 3:**
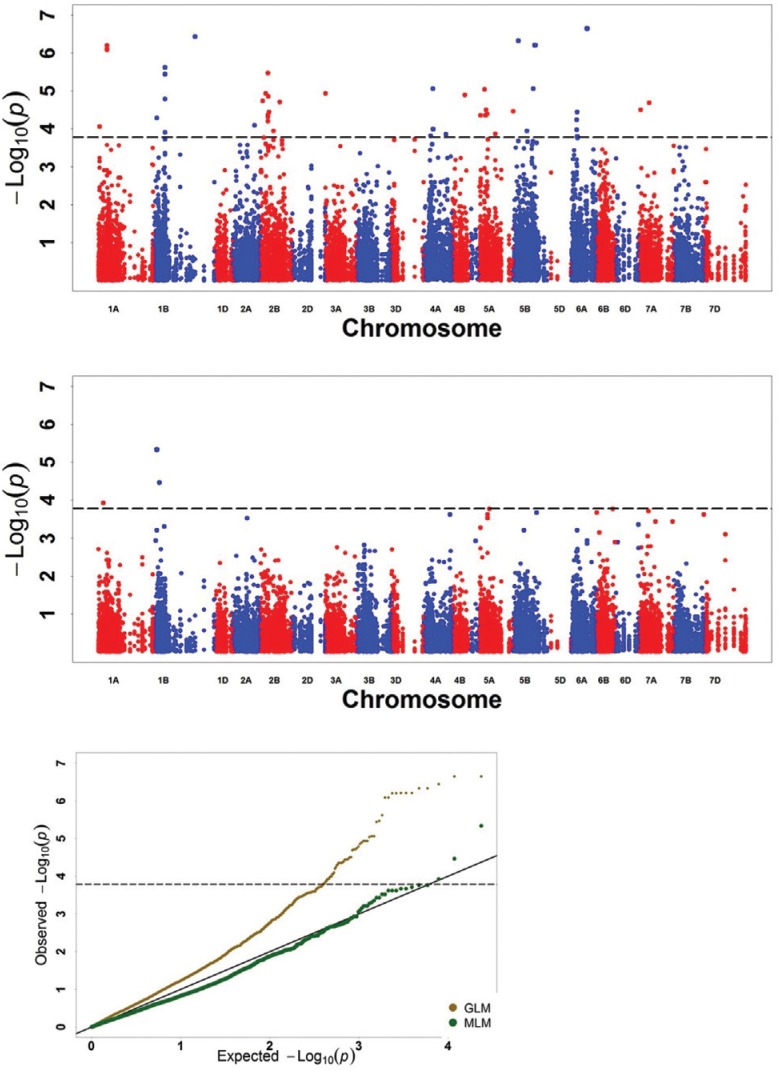
Genome-wide association scan for pre-harvest sprouting resistance in five environments. Manhattan plots for chromosomes carrying significant markers detected by general linear (GLM) and mixed linear (MLM) models; *p*-values converted into –log_10_^(*p*)^ thresholds of 3.782 are indicated by horizontal dashed lines. The Q-Q plot showing the expected null distribution of *p* values assuming no association are represented as a solid black line; *p* values observed using GLM are represented as a brown plot; *p*-values observed using MLM are represented as a dark green plot. a: GLM results; b: MLM results; c: Q-Q plots of GLM and MLM.

In the present study, the Bonferroni-corrected threshold (–log p > 3.782; α = 1) was used as a cutoff to identify MTAs. Significantly associated loci distributed on 12 chromosomes (Figure 3; Table S1) were detected by both the GLM and MLM in the five environments. The GLM identified more markers than the MLM. One marker was detected in both 12WJ and 14CZ. Two significant markers (*tPt-7980* and *wPt-6457*) were detected by both models (Tables 4 and S1; Figure 3), and the two QTL associations with *tPt-7980* and *wPt-6457*, located on Chr. 1B, explained 37% and 30% of the variation, respectively. Based on the mean and BLUP values, eight significant markers were detected. However, no significant markers were detected in all environments. PHS is a complex genetic trait significantly affected by the environment, and maturation at different times under different climatic conditions can affect seed development and confound the phenotype (Kulwal *et al.*, 2012). Thus, it is difficult to detect significant markers in all tested environmental conditions. In the present study, both *tPt-7980* and *wPt-6457* were significant in the GLM and MLM. Thus, further studies are needed to investigate the application of the two markers in breeding programs through marker-assisted selection.

Based on linkage mapping, Munkvold *et al.* (2009) detected two QTL (one DArT and one SSR marker) on Chr. 1B in three environments, accounting for 7% and 4% of the variation, respectively. Singh *et al.* (2014) reported the QTL *Qphs.spa-1B* associated with the markers *tPt-8831, wPt*-*4605*, and *wPt*-*3582* in different environments and located on Chr. 1BS (104.00 cM) based on the Triticarte consensus map 3.0, a chromosomal position similar to that of *wPt-6457* (Table 4). The QTL *Qphs.spa-1A* was also reported by Singh *et al.* (2014) and might be the same with *wPt-668205* that detected in our study, since the distance between the two QTL was less than 1 cM based on the Triticarte consensus map 3.0 (Table 4). Kulwal *et al.* (2012) detected one QTL associated with the marker *wPt-666564* and located on Chr. 1BS based on the Triticarte consensus map 3.0, but it was distant from *wPt-6457* and *tPt-7980* identified in the present study. Due to the incomplete wheat reference genome, we assumed that *wPt-6457* was a previously reported QTL, whereas *tPt-7980* might be a novel QTL.

In the present study, the GLM identified more markers than the MLM. The MLM can reduce the probability of false positives and the Type 1 error in association mapping (Yu *et al.*, 2006). The quantile-quantile (Q-Q) plot also showed that the MLM was more conservative than GLM (Figure 3). However, some significant associations may not be detected using only the MLM (false negatives), because they do not pass the false discovery rate criteria. Thus, we run a BLAST search using all the significant markers sequence from both models and listed the best hit genes.

Previous studies revealed that Chr. 3 contains the *R* genes and the vivipary gene series *TaVp1* (orthologs of maize genes) and *Vp1*, which are involved in germination and are possibly related to PHS resistance (Bailey *et al.*, 1999; Kulwal *et al.*, 2005; Imtiaz *et al.*, 2008), whereas Chr. 4A contains *Phs* 1 (Torada *et al.*, 2008). In present study, we found some candidate genes putatively linked to PHS resistance and divided them into five groups based on the types of encoding proteins. Among these genes, *vp1D* and *AIP2-1* that have been reported to be related to PHS resistance (McKibbin *et al.*, 2002; Gao *et al.*, 2014) were identified using the sequences of significant markers by the GLM, but not by the MLM. The gene *vp1D* involved in germination hence related to PHS resistance (Bailey *et al.*, 1999; Kulwal *et al.*, 2005; Imtiaz *et al.*, 2008). The wheat *AIP2*s could negatively regulate the ABA signaling pathway and play important roles in seed germination, and thus wheat PHS resistance. In addition, other candidate genes, encoding enzymes, regulatory proteins, protection proteins, transport proteins, and other proteins were identified and might regulate or control PHS resistance. Hoshino *et al.* (1989) found that a large vernalization requirement delays germination in winter wheat areas where the late wheat is subjected to ear sprouting by monsoon rain. Cao *et al.* (2016) also detected a QTL associated with PHS resistance on the short arm of Chr. 7B where *Vrn-B3* is located. Therefore, vernalization genes may be involved in the regulation of PHS resistance. In the GLM, we also found the significant marker *wsnp_CAP11_rep_c6622_3044459* located on Chr. 7BS linked to the gene *VRN-3* (*Vrn-B3*) (Table S1), which may be the same QTL with that reported previously. Overall, our data provided a basis for elucidating the genetic mechanisms of PHS resistance in Chinese wheat founder parents.

## Conclusion

We performed a genome-wide association study of pre-harvest sprouting resistance among 80 Chinese wheat founder parents using 6,057 markers. The LD decay distances *(r* ^2^ = 0.3) were approximately 1.94 cM for the whole genome and 5.73, 1.42, 17.48 cM for the A, B, and D genomes, respectively. Two significant marker-trait associations were detected using the GLM and MLM. Twenty-two candidate genes that might control PHS resistance were identified at or near significant loci. The significantly associated loci identified in this study are potential candidates for cloning genes related to PHS resistance and may be used in wheat breeding programs.

## Supplementary material

The following online material is available for this article:

Table S1 - Significant markers in GLM and MLM

Figure S1 - Manhattan plots for 12WJ

Figure S2 - Manhattan plots for 12YA

Figure S3 - Manhattan plots for 13WJ

Figure S4 - Manhattan plots for 13YA

Figure S5 - Manhattan plots for 14CZ

Figure S6 - Manhattan plots for BLUPs

Figure S7 - Manhattan plots for mean values
