# Supplementary material for: Genome-wide association study of pre-harvest sprouting resistance in Chinese wheat founder parents
Source: Genet Mol Biol. 2017 Jul 10;40(3):620–9. doi: 10.1590/1678-4685-GMB-2016-0207 (PMC5596365; doi:10.1590/1678-4685-GMB-2016-0207)
Supplement: Supplementary file 1 [file 1415-4757-gmb-1678-4685-GMB-2016-0207-Suppl01.pdf]

Table S1 - Markers showing significant associations with pre-harvest sprouting resistance in general linear model (GLM) and mixed liner model (MLM).

No.	Marker[a]	Chr.(IWGSC)[b]	Chr.[c]	Locus[c] (cM)	Genes best hit	Genebank	Reference
1	wPt-7905	/	1A	10.75	/		/
2	wPt-8261	1BS	1A	10.75	/		/
3	wsnp_Ex_c4605_8240189	1AL	1A	74.51	/		/
4	wsnp_Ex_c6817_11762714	1AL	1A	74.51	/		/
5	wsnp_Ex_rep_c101414_86780996	1AL	1A	74.51	/		/
6	wsnp_Ku_c4413_8008008	1AL	1A	74.51	/		/
7	wPt-668205	/	1A	473.36	/		/
8	wsnp_Ex_c1429_2745237	1DL	1B	33.39	*Acc-2*	EU660895.1	Chalupska *et al.*, 2008
9	wsnp_Ex_rep_c67747_66422078	1BL	1B	102.36	/		/
10	wsnp_Ex_rep_c67747_66422973	1BL	1B	102.36	/		/
11	rPt-6965	6DS	1B	104.00	*Ty3*	AY040832.1	Hudakova *et al.*, 2001
12	tPt-7918	/	1B	104.00	/		/
13	wPt-6457	1BS	1B	104.00	*Acc-1*	EU660901.1	Chalupska *et al.*, 2008
14	wPt-6457	1BS	1B	104.00	*Acc-1*	EU660901.1	Chalupska *et al.*, 2008
15	tPt-7980	1BS	1B	365.57	/		/
16	tPt-7980	1BS	1B	365.57	/		/
17	wsnp_Ra_c107797_91270622	2AL	2A	61.72	*AIP2-1*	FJ643532.1	Gao *et al.*, (Unpublished)
18	wsnp_JD_c15127_14676522	2AL	2A	186.14	*Rht-B*	KF282628.1	Wu *et al.*, 2013
19	wPt-3949	2BS	2B	18.53	*vp1D*	AJ400714.1	McKibbin *et al.*, 2002
20	wsnp_Ex_c14711_22788263	2BS	2B	43.99	/		/
21	wsnp_Ex_c14711_22788586	2BS	2B	43.99	/		/
22	wPt-1489	/	2B	61.54	/		/
23	wPt-9423	/	2B	61.54	/		/
24	wPt-4301	/	2B	62.26	/		/
25	wPt-3561	2BS	2B	66.55	*Rht-A*	KF282629.1	Wu *et al.*, 2013
26	wsnp_Ex_c13351_21042379	2BS	2B	72.76	/		/
27	wsnp_Ra_c16822_25566950	2BS	2B	72.76	*Rht-A*	KF282629.1	Wu *et al.*, 2013
28	wsnp_Ex_c3044_5620102	2BS	2B	112.35	*NP30_C3*	JN258642.1	Zang *et al.*, 2011
29	wsnp_Ex_c46576_52042185	2BL	2B	166.58	*HAK11*	AJ427980.1	Banuelos *et al.*, 2002

No.	Marker	Chr.(IWGSC)[b]	Chr.[c]	Locus[c] (cM)	Genes best hit	Genebank	Reference
30	wsnp_JD_c47318_32176833	2BL	2B	166.58	*HAK11*	AJ427980.1	Banuelos *et al.*, 2002
31	wsnp_Ex_c9485_15724984	3AS	3A	2.48	*VRN-A1*	KT696536.1	Ivanicova *et al.*, 2016
32	wPt-730263	/	3D	206.25	/		/
33	wsnp_Ex_c8131_13754852	4AS	4A	61.12	*PRR73*	JQ791230.1	Cockram 2012. et al
34	wsnp_Ex_c21383_30513824	4AL	4A	81.90	*Acc-2*	EU660895.1	Chalupska *et al.*, 2008
35	wsnp_Ku_c45197_52288542	4AL	4A	81.90	/		/
36	wsnp_JD_c8309_9321723	4AL	4A	82.47	*Acc-2*	EU660893.1	Chalupska *et al.*, 2008
37	wsnp_Ex_c7280_12498193	4AL	4A	193.19	*waxy gene*	AB272097.1	Takeuchi *et al.* (Unpublished)
38	wsnp_Ex_c7362_12622736	4BS	4B	25.60	*VRN-B1*	KR816810.1	Guedira *et al.* (Unpublished)
39	wsnp_Ex_rep_c67136_65617520	4BL	4B	108.15	*NP35_C3*	JN258646.1	Zang *et al.*, 2011
40	wsnp_Ku_c48254_54492007	5AS	5A	11.22	*starch synthase I gene*	AF091802.1	Li *et al.*, 1999
41	wsnp_Ex_c19892_28910730	5AS	5A	44.09	*AACT1*	GQ403694.1	Silva-Navas *et al.*, 2012
42	wsnp_Ex_rep_c68117_66883366	5AS	5A	52.21	/		/
43	wsnp_Ex_c16317_24795290	5AL	5A	53.86	*UBA1*	M55604.1	Hatfield *et al.*, 1990
44	wsnp_Ex_c19820_28829623	5AL	5A	55.85	*CAC3*	AF056970.1	Ke *et al.*, 2000
45	wPt-1165	/	5A	58.72	/		/
46	wsnp_BE497820A_Ta_2_2	5AL	5A	67.95	/		/
47	wPt-4351	/	5A	294.62	/		/
48	wsnp_Ra_c13424_21239986	5BS	5B	44.40	/		/
49	wsnp_Ra_c13424_21239985	5BS	5B	44.63	/		/
50	wsnp_Ex_rep_c70120_69069789	5BL	5B	117.38	/		/
51	wsnp_Ex_c53426_56666554	5BL	5B	174.16	/		/
52	wsnp_Ku_c11138_18252461	5BL	5B	187.07	*Adf2*	EU878778.1	Brueggeman *et al.*,2008
53	wsnp_Ex_c46494_51987109	5BL	5B	193.79	/		/
54	wsnp_Ku_c1045_2115866	5BL	5B	194.03	/		/
55	wPt-732355	/	6A	46.51	/		/
56	wPt-2822	6AS	6A	46.75	/		/
57	wPt-731010	/	6A	51.26	/		/
58	wPt-7623	/	6A	51.26	/		/

No.	Marker	Chr.(IWGSC)[b]	Chr.[c]	Locus[c] (cM)	Genes best hit	Genebank	Reference
59	wPt-3965	/	6A	53.50	/		/
60	wsnp_Ex_c965_1845447	6AL	6A	138.62	*GRMZM2G043657-like gene*	JQ887933.1	Jiao *et al.*, 2012
61	wsnp_Ku_c9763_16287132	6AL	6A	138.62	*GRMZM2G043657-like gene*	JQ887933.1	Jiao *et al.*, 2012
62	tPt-9948	/	7A	12.99	/		/
63	wPt-9314	7AS	7A	89.52	/		/
64	wsnp_Ex_c7489_12810235	4AL	/	/	/		/
65	wPt-742401	/	/	/	/		/
66	wsnp_Ex_c2066_3877373	2BL	/	/	/		/
67	wsnp_Ex_c43009_49439922	7AS	/	/	/		/
68	wsnp_Ex_c5978_10478584	5AL	/	/	*Acc-2*	EU660895.1	Chalupska *et al.*, 2008
69	wsnp_BE404354B_Ta_2_1	/	/	/	/		/
70	wsnp_CAP11_rep_c6622_3044459	7BS	/	/	*VRN3 (vrn-B3)*	DQ890165.1	Yan *et al.*, 2006
71	wsnp_Ex_c35195_43389213	/	/	/	/		/
72	wsnp_Ex_c19260_28187434	2AS	/	/	*Acc-1*	EU660901.1	Chalupska *et al.*, 2008
73	wsnp_JD_c15127_14676999	/	/	/	/		/
74	wPt-667560	/	/	/	/		/
75	wsnp_CAP11_c1737_946813	2AL	/	/	*Hox-1*	FJ477091.1	Wicker *et al.*, 2009
76	wsnp_Ra_c24707_34262900	5AS	/	/	/		/

No.	DEFINITION
1	/
2	/
3	/
4	/
5	/
6	/
7	/
8	Triticum aestivum clone BAC 1825J10 cytosolic acetyl-CoA carboxylase (Acc-2) and putative amino acid permeases genes
9	/
10	/
11	Hordeum vulgare Ty3/gypsy retrotransposon cereba gag-pol polyprotein gene
12	/
13	Triticum aestivum clone BAC 198E19 plastid acetyl-CoA carboxylase(Acc-1) gene, nuclear gene for plastid product.
14	Triticum aestivum clone BAC 198E19 plastid acetyl-CoA carboxylase(Acc-1) gene, nuclear gene for plastid product.
15	/
16	/
17	Triticum aestivum ABI3-interacting protein 2-1 protein (AIP2-1) gene
18	Triticum durum cultivar Langdon clone BAC 315P18 chromosome 4B DELLA protein (Rht-B) gene
19	Triticum aestivum vp1D gene for VIVIPAROUS1
20	/
21	/
22	/
23	/
24	/
25	Triticum aestivum cultivar Chinese Spring clone BAC 351D1 chromosome 4A DELLA protein (Rht-A) gene
26	/
27	Triticum aestivum cultivar Chinese Spring clone BAC 351D1 chromosome 4A DELLA protein (Rht-A) gene
28	Oryza eichingeri clone NP30_C3 hypothetical protein gene
29	Oryza sativa HAK11 gene for putative potasium transporter

No.	DEFINITION
30	Oryza sativa HAK11 gene for putative potasium transporter
31	Triticum aestivum cultivar Tahti VRN-A1 (VRN-A1) gene
32	/
33	Hordeum vulgare subsp. vulgare cultivar OWB-D PRR73 (PRR73) gene
34	Triticum aestivum clone BAC 1825J10 cytosolic acetyl-CoA carboxylase (Acc-2) and putative amino acid permeases genes
35	/
36	Triticum urartu clone BAC 252P12+402A6 cytosolic acetyl-CoA carboxylase (Acc-2) and putative amino acid permease genes
37	Triticum aestivum waxy gene for granule-bound starch synthase I
38	Triticum aestivum cultivar Pioneer brand 26R61 (PI612153) VRN-B1 (VRN-B1) gene
39	Oryza eichingeri clone NP35_C3 hypothetical protein gene
40	Aegilops tauschii starch synthase I gene
41	Secale cereale aluminum activated citrate transporter 1 (AACT1) gene
42	/
43	T.aestivum ubiquitin activating enzyme E1 (UBA1) gene
44	Arabidopsis thaliana carboxyltransferase alpha subunit (CAC3) gene
45	/
46	/
47	/
48	/
49	/
50	/
51	/
52	Hordeum vulgare subsp. vulgare Adf2 (Rpg4), RGA1, NBS-LRR-S/TPK stem rust resistance protein (Rpg5), and ADF3 genes
53	/
54	/
55	/
56	/
57	/
58	/

No.	DEFINITION
59	/
60	Zea mays isolate Zheng58 clone 1 GRMZM2G043657-like gene
61	Zea mays isolate Zheng58 clone 1 GRMZM2G043657-like gene
62	/
63	/
64	/
65	/
66	/
67	/
68	Triticum aestivum clone BAC 1825J10 cytosolic acetyl-CoA carboxylase (Acc-2) and putative amino acid permeases genes
69	/
70	Triticum aestivum cultivar CS(Hope7B) VRN3 (vrn-B3) gene
71	/
72	Triticum aestivum clone BAC 198E19 plastid acetyl-CoA carboxylase(Acc-1) gene, nuclear gene for plastid product.
73	/
74	/
75	Hordeum vulgare subsp. vulgare cultivar Haruna Nijo Hox-1 gene
76	/

No.	12WJ		12YA		13WJ		13YA		14CZ		Mean		BLUP	
	-Log(p)	R^2 (%)	-Log(p)	R^2 (%)	-Log(p)	R^2 (%)	-Log(p)	R^2 (%)	-Log(p)	R^2 (%)	-Log(p)	R^2 (%)	-Log(p)	R^2 (%)
1	4.36	22.83												
2	4.44	21.74												
3							4.37	22.89						
4							4.79	24.78						
5							4.06	21.46						
6							5.62	28.39						
7														
8					4.93	21.87								
9							4.36	22.83						
10							4.36	22.83						
11	5.04	25.90												
12	4.36	22.83												
13					4.40	23.01								
14														
15					4.06	21.47								
16														
17											3.79	17.49		
18									3.98	20.08				
19					6.44	27.30								
20									3.80	14.43	5.57	24.39	5.68	25.23
21									5.44	23.76	5.57	24.39	5.68	25.23
22									4.74	21.12				
23									4.31	17.27				
24	4.50	17.16							4.50	20.22	6.54	27.85	6.45	27.99
25									3.98	18.14	5.69	24.85	5.64	25.05
26							3.90	17.84						
27							4.45	20.00						
28							4.69	20.95						
29							4.20	20.01						

No.	12WJ		12YA		13WJ		13YA		14CZ		Mean		BLUP	
	-Log$^{(p)}$	R^2 (%)	-Log$^{(p)}$	R^2 (%)	-Log$^{(p)}$	R^2 (%)	-Log$^{(p)}$	R^2 (%)	-Log$^{(p)}$	R^2 (%)	-Log$^{(p)}$	R^2 (%)	-Log$^{(p)}$	R^2 (%)
30							4.28	19.36						
31	4.94	23.00									4.87	18.69	4.73	18.46
32											3.90	17.96	4.10	19.06
33							4.00	18.21						
34					5.06	22.34								
35					3.94	17.99								
36					3.96	18.07								
37							4.00	18.21						
38											4.07	18.63	4.15	19.25
39							4.47	21.84						
40	6.20	28.84												
41	3.94	19.57												
42	4.41	21.60												
43	6.28	29.15												
44	4.44	21.74												
45					4.12	20.37								
46	6.21	28.87												
47							4.24	20.85						
48							4.16	19.84						
49							3.87	19.26						
50					6.09	28.40								
51					3.85	19.19								
52							4.39	20.77						
53							3.82	19.03						
54							6.65	30.51						
55							4.71	22.88						
56					6.20	28.84								
57					4.90	23.65								
58					3.91	19.41								

No.	12WJ		12YA		13WJ		13YA		14CZ		Mean		BLUP	
	-Log(p)	R^2 (%)	-Log(p)	R^2 (%)	-Log(p)	R^2 (%)	-Log(p)	R^2 (%)	-Log(p)	R^2 (%)	-Log(p)	R^2 (%)	-Log(p)	R^2 (%)
59					3.91	19.41								
60							6.21	28.87						
61							4.10	19.57						
62					6.33	29.32								
63					6.21	28.87								
64			5.06	22.35										
65					6.33	29.32								
66					5.00	22.12								
67					6.09	28.40								
68							4.71	22.88						
69							4.86	22.69						
70							6.65	30.51						
71							5.47	25.09						
72									3.80	14.43	5.21	23.08	5.33	23.90
73									4.94	23.00				
74													3.79	17.79
75													3.85	18.02
76											4.11	18.78	4.52	20.74

Abbreviation: 12WJ, Wenjiang 2012; 12YA, Ya'an, 2012; 13WJ, Wenjiang 2013; 13YA, Ya'an 2013; 14CZ, Chongzhou, 2014; BLUP, the best linear unbiased prediction; Chr., Chromosome; Envir., Environment; GLM, general linear model; MLM, mixed liner model; NO., number order.

[a] significant makers with underline were detected in MLM, others were detected in GLM.

[b] the chromosome information were from International Wheat Genome Sequencing Consortium (IWGSC) database, http://www.wheatgenome.org/.

[c] the Chromosome and position (cM) of DArT markers were from the Triticarte consensus map 3.0 (http://www.triticarte.com.au/) and Chromosome and position (cM) of SNP markers were from Illumina 9K SNP consensus map (Cavanagh *et al.,* 2013).

References

Banuelos MA, Garciadeblas B, Cubero B and Rodríguez-Navarro A (2002) Inventory and functional characterization of the HAK potassium transporters of rice. Plant Physiol 130:784-795.

Brueggeman R, Druka A, Nirmala J, Cavileer T, Drader T, Rostoks N, Mirlohi A, Gill BU, Kudrna D, Whitelaw C, et al. (2008) The stem rust resistance gene Rpg5 encodes a protein with nucleotide-binding-site, leucine-rich, and protein kinase domains. Proc Natl Acad Sci USA 105:14970-14975.

Chalupska D, Lee HY, Faris JD, Evrard A, Chalhoub B, Haselkorn R and Gornicki P (2008) Acc homoeoloci and the evolution of wheat genomes. Proc Natl Acad Sci USA 105:9691-9696.

Cockram J, Thiel T, Steuernagel B, Stein N, Taudien S, Bailey PC and O'Sullivan DM (2012) Genome dynamics explain the evolution of flowering time CCT domain gene families in the Poaceae. PLoS One 7:e45307.

Hatfield PM, Callis J and Vierstra RD (1990) Cloning of ubiquitin activating enzyme from wheat and expression of a functional protein in Escherichia coli. Journal of Biological Chemistry 265:15813-15817.

Hudakova S, Michalek W, Presting GG, ten Hoopen R, dos Santos K, Jasencakova Z and Schubert I (2001) Sequence organization of barley centromeres. Nucleic Acids Res 29:5029-5035.

Ivanicova Z, Jakobson I and Reis D (2016) Characterization of new allele influencing flowering time in bread wheat introgressed from Triticum militinae. New Biotechnology 33:718-727.

Jiao Y, Zhao H, Ren L, Song W, Zeng B, Guo J, Wang B, Liu Z, Chen J, Li Wei, et al. (2012) Genome-wide genetic changes during modern breeding of maize. Nature Genet 44:812-815.

Ke J, Wen TN, Nikolau BJ and Wurtele ES (2000) Coordinate regulation of the nuclear and plastidic genes coding for the subunits of the heteromeric acetyl-coenzyme A carboxylase. Plant Physiol 122:1057-1072.

Li Z, Rahman S, Kosar-Hashemi B, Mouille G, Appels R and Morell MK (1999) Cloning and characterization of a gene encoding wheat starch synthase I. Theor Appl Genet 98:1208-1216.

McKibbin RS, Wilkinson MD, Bailey PC, Flintham JE, Andrew LM, Lazzeri PA, Gale MD, Lenton JR and Holdsworth (2002) Transcripts of Vp-1 homeologues are misspliced in modern wheat and ancestral species. Proc Natl Acad Sci USA 99:10203-10208.

Silva-Navas J, Benito C, Tellez-Robledo B, Yang GP, Zhang Q and Allard RW (2012) The ScAACT1 (ScMATE) gene at the Qalt5 locus contributes to aluminum tolerance in the rye Secale cereale L. Mol breed 30:845-856.

Wicker T, Krattinger SG, Lagudah ES, Komatsuda T, Pourkheirandish M, Matsumoto T, Cloutier S, Reiser L, Kanamori H, Sato K, et al. (2009) Analysis of intraspecies diversity in wheat and barley genomes identifies breakpoints of ancient haplotypes and provides insight into the structure of diploid and hexaploid Triticeae gene pools. Plant Physiol 149:258-270.

Wu J, Kong X, Shi C, Gu Y, Jin C, Gao L and Jia J (2013) Dynamic evolution of Rht-1 homologous regions in grass genomes. PloS one 8:e75544.

Yan L, Fu D, Li C, Blechl A, Tranquilli G, Bonafede M, Sanchez A, Valarik M, Yasuda S, Dubcovsky J, et al. (2006) The wheat and barley vernalization gene VRN3 is an orthologue of FT. Proc Natl Acad Sci USA 103:19581-19586.

Zang LL, Zou XH, Zhang FM, Yang Z and Song GE (2011) Phylogeny and species delimitation of the C-genome diploid species in Oryza. J Syst Evol 49:386-395.
