# Supplementary material for: Genome-wide association study of pre-harvest sprouting resistance in Chinese wheat founder parents
Source: Genet Mol Biol. 2017 Jul 10;40(3):620–9. doi: 10.1590/1678-4685-GMB-2016-0207 (PMC5596365; doi:10.1590/1678-4685-GMB-2016-0207)

Figure S2 - Genome-wide association scan for pre-harvest sprouting resistance in Ya'an in 2012. Manhattan plots for chromosomes carrying significant markers detected by general linear (GLM) and mixed linear (MLM) models; *p*-values converted into $-\log_{10}(p)$ thresholds of 3.782 are indicated by horizontal dashed lines.

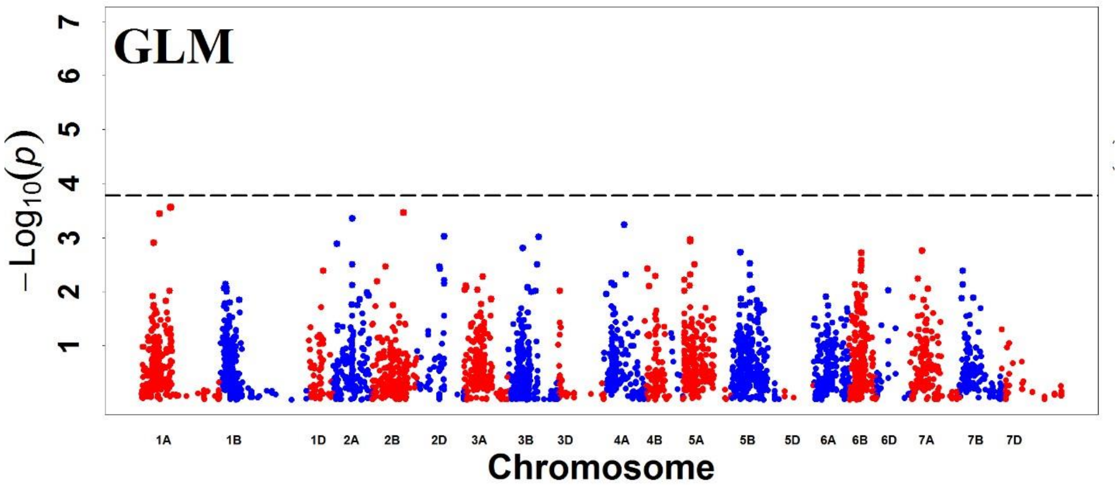

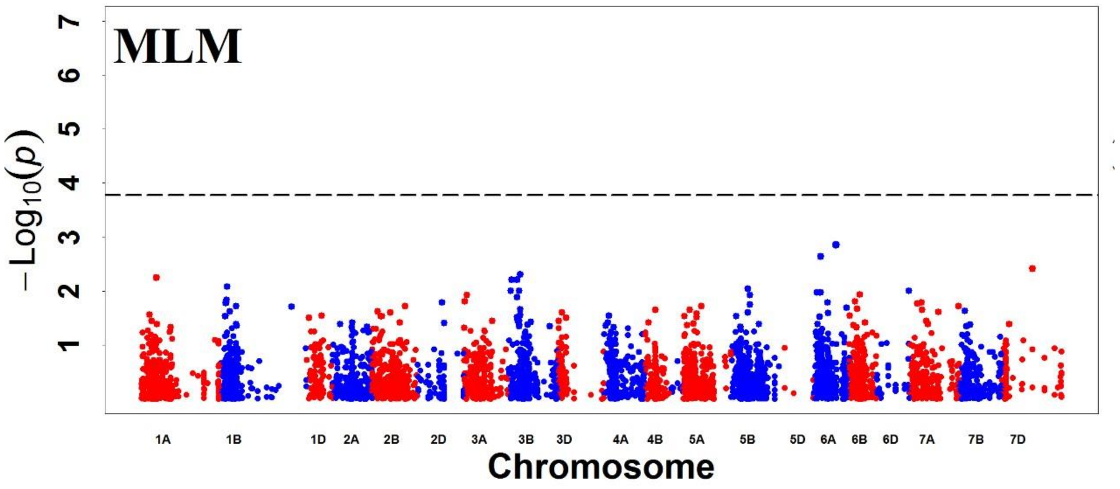

Supplement: Figure S2 [file 1415-4757-gmb-1678-4685-GMB-2016-0207-Suppl03.pdf]
